# The rootstock genotype shapes the diversity of pecan (*Carya illinoinensis*) rhizosphere microbial community

**DOI:** 10.3389/fmicb.2024.1461685

**Published:** 2024-10-03

**Authors:** Wei Ren, Lu Zhang, Braden Tondre, Xinwang Wang, Tingying Xu

**Affiliations:** ^1^Boone Pickens School of Geology, Oklahoma State University, Stillwater, OK, United States; ^2^Department of Horticulture and Landscape Architecture, Oklahoma State University, Stillwater, OK, United States; ^3^USDA-ARS, Southern Plains Agricultural Research Center, Pecan Breeding and Genetics, College Stations, TX, United States

**Keywords:** grafting, rhizosphere microbes, core microbiome, mycorrhizal fungi, microbial community

## Abstract

Pecans (*Carya illinoinensis*), one of the most valuable native North American nut crops, are commonly propagated through grafting to preserve the desired characteristics from parent trees. Since successful cultivation of pecan trees relies on the interplay among scion varieties, rootstocks, and soil conditions, this study investigated the microbial change to communities in the soils and roots of southern (87MX5-1.7) and northern (Peruque) rootstocks in a rootstock test orchard. Both grafted with the ‘Pawnee’ scion cultivar. Bacterial 16S ribosomal RNA and fungal ITS were amplified from both roots and rhizosphere soils of the two 10-year-grafted trees, then sequenced and annotated into trophic and nutrient-related groups to characterize the rhizosphere microbiota. The Peruque roots had a higher relative abundance of saprotroph fungi, while 87MX5-1.7 exhibited higher levels of symbiotroph fungi and nitrogen fixation-related bacteria. Among them, the presence of symbiotroph fungi, particularly ectomycorrhizal fungi, notably differed between these two rootstocks, with a significantly higher presence observed in the root of 87MX5-1.7 compared to Peruque. This variation likely leads to divergent pathways of nutrient translocation: Peruque was in favor of multiple fungi (*Russula* and *Inocybe*) to gain nutrition, while 87MX5-1.7 preferred a specific domain of fungi (*Tuber*) and nitrogen fixation-related bacteria (*Bradyrhizobia*) to form beneficial symbiosis. Moreover, the presence of pathogens suggested a potential risk of Fusarium patch and snow molds in 87MX5-1.7, while canker and black foot disease pose threats in Peruque. The findings of this study suggest that rootstocks from different origins shape rhizosphere microbes differently, potentially affecting nutrient uptake and nut yield. Exploring rootstock-microbe combinations could provide insights into optimizing scion growth and ultimately increasing nut yield. By understanding how different rootstock-microbe interactions influence pecan tree development, growers can strategically select combinations that promote beneficial symbiotic relationships, enhancing nutrient uptake, disease resistance, and overall tree vigor.

## 1 Introduction

Pecan (*Carya illinoinensis*) is the most valuable native North American nut crop, spanning a broad distribution from northern Illinois, USA, to the south of Zaachila, Oaxaca, Mexico (Thompson and Grauke, 1991). Pecan trees demonstrate remarkable adaptation to diverse environments, including various soil types and climate conditions (Thompson and Grauke, 1991). Native pecan trees have a long lifespan that can extend hundreds of years (Randall et al., 2021; Thompson and Grauke, 1991). Pecans are now extensively cultivated in subtropical plantation areas due to their high-quality nut production (Storey et al., 1995). However, transplanting well-established cultivars without compromising nut yield poses significant challenges. Moreover, the relocation of pecan cultivars/rootstocks from their native regions to new plantation areas can lead to changes in the soil microbial communities surrounding them. These alternations may arise from differences in geographical climates, extended lifespans, and hybridity among species (Cervantes et al., 2023; Randall et al., 2021). These challenges underscore the importance of selecting appropriate rootstock varieties to ensure successful transplantation and optimal tree performance.

Successful cultivation of pecan trees is governed by various factors, including scion varieties, rootstocks, and soil environments (Gast and Overcash, 1980; Grauke and Thompson, 1995). Notably, selecting an appropriate rootstock has been recognized as a crucial determinant of scion performance, impacting various aspects such as plant height, trunk diameter, canopy width, flowering time, nut yield, nut quality, disease resistance, and insect resistance (Doornbos et al., 2011; Fallahi et al., 2001; Grauke and Thompson, 1995; Turner et al., 2013; Wood, 2017). The rhizosphere, acting as an interface between plant roots and soil while hosting abundant microbes, forms a narrow zone surrounding the roots within the soil (Bell et al., 2015; Kumar and Dubey, 2020). These microbial communities in the rhizosphere are closely associated with plant nutrient uptake and growth (Tedersoo et al., 2020; Yin et al., 2021), with their composition being influenced by plant genotypes (Mendes et al., 2013). Studies have shown that pecan soil microbial communities were influenced by the seedstock origin (Cervantes et al., 2023). The soil microbiome is a diverse community, and its composition can be influenced by factors such as agricultural practices, soil type, and climate (Berg et al., 2014). Research has demonstrated the crucial role of soil microorganisms in nutrient cycling, disease suppression, and overall soil health, as well as their interactions with the root system significantly impact plant growth and productivity (Berg et al., 2014; Mendes et al., 2013). A balanced and diverse soil microbiome, in particular, contributes to disease suppression and optimal plant health in pecan orchards (Cervantes et al., 2023; Randall et al., 2021).

Within the soil microbial community, certain beneficial fungi inhabiting the plant rhizosphere, such as mycorrhizal fungi, establish mutualistic symbiosis with plant roots, thereby sustaining the health of the host plant (Bonfante and Genre, 2010; Field et al., 2019; Smith and Read, 2008). Uniquely, pecan trees lack root hairs (Woodroof and Woodroof, 1934) and heavily rely on mycorrhizal fungi to expand their nutrient absorption area, effectively substituting for root hairs (Ortas, 2018). Moreover, some rhizosphere microorganisms possess the potential for both nitrification and denitrification, playing a crucial role in the nitrogen cycle within the plant rhizosphere (Philippot et al., 2008). Furthermore, certain beneficial microorganisms have been identified as key contributors to defense against soilborne pathogens (Mendes et al., 2013), which serve as a natural shield for pecan rootstocks and help maintain soil health. Consequently, the connection between soil microorganisms and pecan rootstocks presents a promising opportunity for sustainable and resilient orchard management, fostering healthier pecan trees, increased yields, and a more sustainable future of pecan cultivation. Despite the widespread recognition of the rootstock’s influence on scion performance (Friesen et al., 2011; Randall et al., 2021; Turner et al., 2013), the specific mechanisms through which rootstocks modulate soil microbial communities and subsequently affect scion growth are not well understood. Additionally, the intricate relationship between different pecan rootstocks and soil microorganisms remains largely unexplored.

Since 2009, the USDA ARS Pecan Breeding Program has embarked on a project exploring the influence of 12 distinct rootstocks on the growth of the same scion, ‘Pawnee,’ in Somerville, TX. These rootstocks, derived from various geographical locations in Mexico and USA, are either widely used in the USA or identified as potential rootstocks due to their vigorous growth (Wang et al., 2024), tolerance to high pH soil environment (Miyamoto et al., 1985), or resistance to cotton root rot disease (Nesbitt, 1992). Preliminary findings suggest that Pawnee scions grafted onto rootstocks from southern provenances demonstrate accelerated growth, characterized by quicker bud break, earlier flowering, increased plant height, larger trunk diameter and wider canopy width. In contrast, scions grafted onto the rootstocks from northern provenances show slower bud break and flowering, alongside slower overall tree growth (Wang et al., 2024). These observations drive us to further explore the soil microbial communities and their contributions to scion growth. This study aims to elucidate how different pecan rootstocks shape the soil microbial communities and subsequently affect scion growth. Understanding the dynamics of soil microbial community interactions with various pecan rootstocks is essential for optimizing pecan orchard management practices, enhancing sustainability, and mitigating potential challenges associated with soil-borne pathogens and nutrient availability.

Therefore, the primary objectives of this study are: (1) to characterize the soil microbial communities associated with different pecan rootstocks and the core microbiome in pecan rhizosphere; and (2) to investigate the influence of these rootstocks on the composition of pathogenic, symbiotic, and saprotrophic microbes in pecan rhizosphere. Through accomplishing these objectives, valuable insights into the complex interplay among pecan rootstocks, soil microorganisms, and scion growth could be provided. Results from this study will offer a foundation for developing sustainable orchard management practices and enhancing overall pecan production.

## 2 Materials and methods

### 2.1 Rootstock selection

In 2012, five healthy, uniform, 3-year-old seedlings (selected based on height and stem diameter; data not shown) of 12 different rootstocks originating from various provenances (see Supplementary Table S1) were planted in four blocks in a test orchard at the USDA ARS Pecan Breeding Orchard in Somerville, TX. Each block was arranged with four rows, each containing 15 trees, totaling 60 trees per block. Each block included five replicates of each rootstock in an interlocking randomized design. Additionally, five rows with another set of five rootstocks were planted as borders to separate the four blocks. Each of these border rows included 15 trees with three replicates of each rootstock (data not shown). In 2013, the scion cultivar ‘Pawnee’ was grafted onto these seedlings in the four blocks, and ‘Kanza’ was grafted onto the border trees in the same year. Field growth was monitored annually by measuring rootstock and scion diameters, as well as plant height. Plant height was measured in centimeters from the ground to the top of the main stem using a tape measure for trees under 2 meters (before 2017) and with a laser camera (Vertex Laser Geo, Haglöf, Sweden) for taller trees (after 2017) during the dormant season. Rootstock and scion stem diameters were measured in centimeters using a digital caliper.

Field observation revealed that a southern provenance, 87MX1-2.2 and 87MX5-1.7, are the fastest-growing rootstock, while a northern provenance, ‘Peruque’, originated from Peruque, St. Charles, MO, United States, is the slowest-growing rootstock. However, among the five replicates of 87MX1-2.2, one tree exhibited non-uniform growth, appearing smaller than the other four trees. Another southern provenance, 87MX5-1.7, originated from Jaumave, Tamaulipas, Mexico, exhibited uniform and vigorous growth and showed no significant difference compared to 87MX1-2.2 (Supplementary Table S1). Therefore, 87MX5-1.7 and ‘Peruque’ were chosen for further soil sampling purposes.

### 2.2 Sampling and soil elemental analysis

The rhizosphere soil and root sample were collected from the USDA ARS Pecan Breeding Program in Somerville, TX, in 2022. Three pecan trees of 87MX5-1.7 and Peruque were selected for sampling. Subsamples were taken from three directions of each tree and combined as one sample. During sampling, root with soil attached, ranging from a depth of 5 to 20 cm and taken 1 m away from the base of the target tree trunk, were collected and stored in one-gallon Ziploc bags for further DNA extraction and elemental measurement. Two-millimeter meshes were utilized to remove litter, rocks, and other debris from the samples. The fine roots were carefully selected and gently shaken to dislodge the soil attached to them, which was then collected as rhizosphere soil (Berg and Smalla, 2009; Castellano-Hinojosa et al., 2023). Then the fine root was washed with sterilized deionized water, dried by wiping, ground into powder using a mortar and pestle in liquid nitrogen, and then stored at −80°C for further DNA extraction. A portion of the rhizosphere soil samples were also stored at −80°C for further DNA extraction to analyze microbial community. The remaining soil samples were air-dried at room temperature, ground into fine powder using a mortar and pestle, and then sieved with a 0.15 mm mesh for soil elemental analysis.

Total carbon (TC) and total sulfur (TS) in rhizosphere soil were tested using a Carbon/Sulfur Analyzer (CS-2000, ELTRA, Haan, Germany), while total nitrogen was measured using an elemental analyzer (ECS 4010, Costech Analytical Technologies, Inc., Valencia, CA, United States). To determine the concentrations of total potassium (K), calcium (Ca), phosphorus (P), zinc (Zn), magnesium (Mg), iron (Fe), copper (Cu), and other trace elements, soil samples were digested using methods modified from EPA Method 3050 and Adeboye et al. (2020). Briefly, the air-dried soil (0.1 g) was weighed and put into a Teflon container with 10 milliliters HNO_3_ (trace metal grade) and two milliliters H_2_O_2_ (trace metal grade) overnight. Then, the samples were digested using the PicoTrace^®^ pressure digestion apparatus at 170°C for 5 h as Adeboye et al. (2020). The digestion solution was subsequently evaporated at 170°C for 1 h, and deionized water was added to reach a final volume of 10 milliliters for elemental analysis. Elemental analysis of digestion solutions was conducted on Inductively Coupled Plasma Optical Emission spectroscopy (ICP-OES, iCAP PRO XPS DUO, Thermo Scientific Inc., Waltham, MA, United States).

### 2.3 Microbial DNA sequencing

The total microbial DNA from root and soil samples was extracted using Qiagen DNeasy Plant Pro Kit and Qiagen DNeasy PowerSoil Pro Kit, respectively (Qiagen, Hilden, Germany). The extracted DNA was transported on dry ice to the University of Minnesota Genomics Center (UMGC) for library preparation and amplicon sequencing. To analyze the bacterial community, the primer pairs 515F (5′-GTGCCAGCMGCCGCGGTAA-3′) and 806R (5′-GGACTACHVGGGTWTCTAAT-3′) were used to amplify the V4 region of bacterial 16S rDNA (Gohl et al., 2016). For fungal community, the ITS2 (internal transcribed spacer 2) region of fungi was amplified using the primer pairs fITS7 (5′-GTGARTCATCGAATCTTTG-3′) and ITS4 (5′-TCCTCCGCTTATTGATATGC-3′) (Nilsson et al., 2019). Sequencing was conducted using a paired-end method with a MiSeq 2 × 300 cycle v3 kit (Illumina, San Diego, CA, United States). The raw amplicon data files in this study have been submitted to National Center for Biotechnology Information (NCBI) under BioProject PRJNA1102032 (bacterial 16S rDNA: SAMN41011636-SAMN41011647; fungal ITS2: SAMN41011648-SAMN41011659).

### 2.4 Bioinformatics analysis

Mothur (v1.47) was utilized for the processing of bacterial 16S rRNA and fungal ITS sequences (Schloss et al., 2009) following the standard operating procedure^1^ and the method described by Kozich et al. (2013). The paired raw data from MiSeq were assembled and generate the fasta files in Mothur. For bacterial 16S rRNA, sequences containing ambiguous bases, two or more mismatches to the primer, homopolymers exceeding 8 bp in length, or with a length exceeding 325 bp were filtered out in Mothur. Chimeric sequences were then eliminated using the VSEARCH (v2.21.1) (Rognes et al., 2016). The remaining 16S rRNA sequences were aligned to the Mothur-formatted SILVA v138.1 seed database (downloaded from https://mothur.org/wiki/silva_reference_files/ on October 17, 2022), following the protocol outlined by Wang et al. (2007) in Mothur. Similarly, fungal ITS sequences shorter than 300 bp or longer than 590 bp were excluded and the remaining ITS sequences were aligned to the Mothur-formatted dynamic UNITE+INSD database (download from https://mothur.org/wiki/unite_its_database/ on Oct 17, 2022) according to Wang et al. (2007) in Mothur. The unique reads of both 16S RNA and ITS were clustered into operational taxonomic units (OTUs) with 97% similarity (Cervantes et al., 2022a), separately. The groups of Archaea, Animalia, and Plantae were removed in Mothur. The bacterial and fungal taxa were classified into phylum, class, order, family, and genus to acquire the unique performance of taxa across two rootstocks. The relative abundance of different taxa was calculated by dividing the count of each item by the total counts in R (v4.3.2) with the R package “tidyverse (v 2.0).” The OTUs with low average relative abundance were defined as “other” with the specific limit value provided alongside the related figures.

To differentiate the unique OTUs of two rootstocks, R package “VennDiagram (v1.7.3)” (Chen, 2022) was utilized. In Venn analysis, the positive value of the average counts of fungal and bacterial OTUs was defined as “TRUE” to indicate the presence of these OTUs in this treatment. The list of overlapping OTUs was output and annotated with functional information, followed by calculating relative abundance of functional groups in Excel. Chao1, Richness, Shannon, and inverse of Simpson (invSimpson) indices were calculated to assess *α*-diversity by Mothur, following the method in the standard operating procedure in Mothur website. Principal Coordinate Analysis (PCoA) using Bray–Curtis dissimilarity metric in Mothur was performed to display the difference among treatments (Bray and Curtis, 1957; Legendre and Gallagher, 2001). The first two axes were then plotted using R package “ggplot2 (v3.5).”

To taxonomically parse OTUs, functional groups were defined using FUNGuild (v1.0) and FAPROTAX (v1.2.6) following the methods as Louca et al. (2016) and Nguyen et al. (2016). FUNGuild categorized fungal taxa according to their trophic modes, such as saprotrophs, pathogens, and symbiotrophs. Furthermore, it provided additional detailed ecological guilds including plant pathogens, ectomycorrhizal (ECM) fungi, arbuscular mycorrhizal (AM) fungi, and others. These detailed guild assignments offer insights into the specific ecological roles and functions of fungal taxa within ecosystems. In FAPROTAX, bacteria were annotated based on their roles in biogeochemistry cycles, such as nitrogen fixation, respiration, nitrification, denitrification, anammox, and ammonification (Louca et al., 2016).

### 2.5 Statistical analysis

In this study, SPSS 26.0 (IBM Corporation, Chicago, United States), was used for conducting ANOVA (analysis of variance), between-subject effect tests, and *post hoc* multiple comparisons. Between-subject effect tests were used to analysis the contribution of sample types (root and rhizosphere soil) and genotypes of rootstock (Peruque and 87MX5-1.7). Duncan’s test at a significance level of 0.05 was used to assess statistically significant differences (*N* = 3). PCoA (principal coordinates analysis) and AMOVA (analysis of molecular variance) were performed using Mothur. The Venn plot, PCA plot, and boxplot were generated using R version 4.3.2 (R Foundation for Statistical Computing, Vienna, Austria). Origin Pro 2024 (OriginLab, Northampton, MA, United States) was used to generate the stacked plot, heatmap, and pie chart for functional group analysis.

## 3 Results

### 3.1 Soil information

The general rhizosphere soil chemical properties of *C. illinoinensis* cv. ‘Pawnee’ grafted on two different rootstocks is presented in Supplementary Table S2. The major nutrient concentrations did not show significant differences between the two rootstocks, 87MX5-1.7 and Peruque.

### 3.2 Effect of genotype of rootstock on the microbial number and diversity

**Figure 1:**
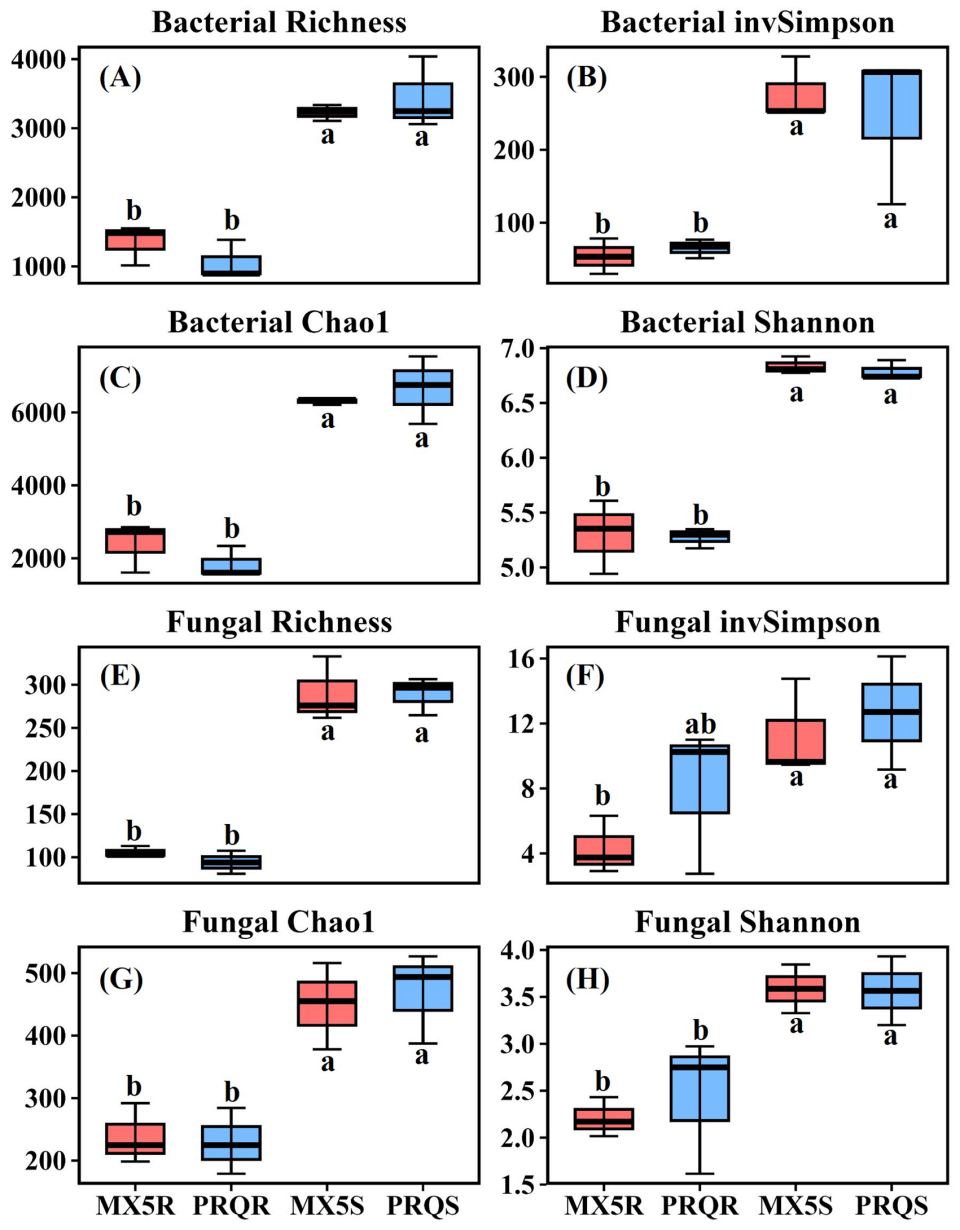
The bacterial diversity indices of Richness **(A)**, inverse of Simpson **(B)**, Chao1 **(C)**, and Shannon **(D)** in the root and rhizosphere soil of pecan grown with different rootstocks. The fungal diversity indices of Richness **(E)**, inverse of Simpson **(F)**, Chao1 **(G)**, and Shannon **(H)** in the root and rhizosphere soil of pecan grown with different rootstocks. Different lowercase letters in each graph indicate a significant difference among the means by Duncan’s test (*p* < 0.05), and the same letter means no significant difference among the means by Duncan’s test (*p* > 0.05). PRQS = soil samples of pecan with rootstock Peruque; MX5S = soil sample of pecan with rootstock 87MX5-1.7; PRQR = root sample of pecan with rootstock Peruque; MX5R = root sample of pecan with rootstock 87MX5-1.7.

To characterize the microbial communities which were present in the rhizosphere soil and roots of two pecan rootstocks, fungal ITS and bacterial 16S genes were amplified and then sequenced. A total of 780,772 reads were obtained from the rhizosphere soil and roots of the two different rootstocks. After filtering out the low-quality reads, the sequences were classified into 2,988 fungal OTUs and 10,722 bacterial OTUs. The fungal and bacterial *α*-diversity indices in both the roots and rhizosphere soil exhibited no significant differences between the two rootstocks (Figure 1). However, within each index, there was consistently a significant difference between the root and the rhizosphere soil samples, with the rhizosphere soil being significantly higher than the root in richness, invSimpson, Chao1, and Shannon indices of both fungal and bacterial communities.

**Figure 2:**
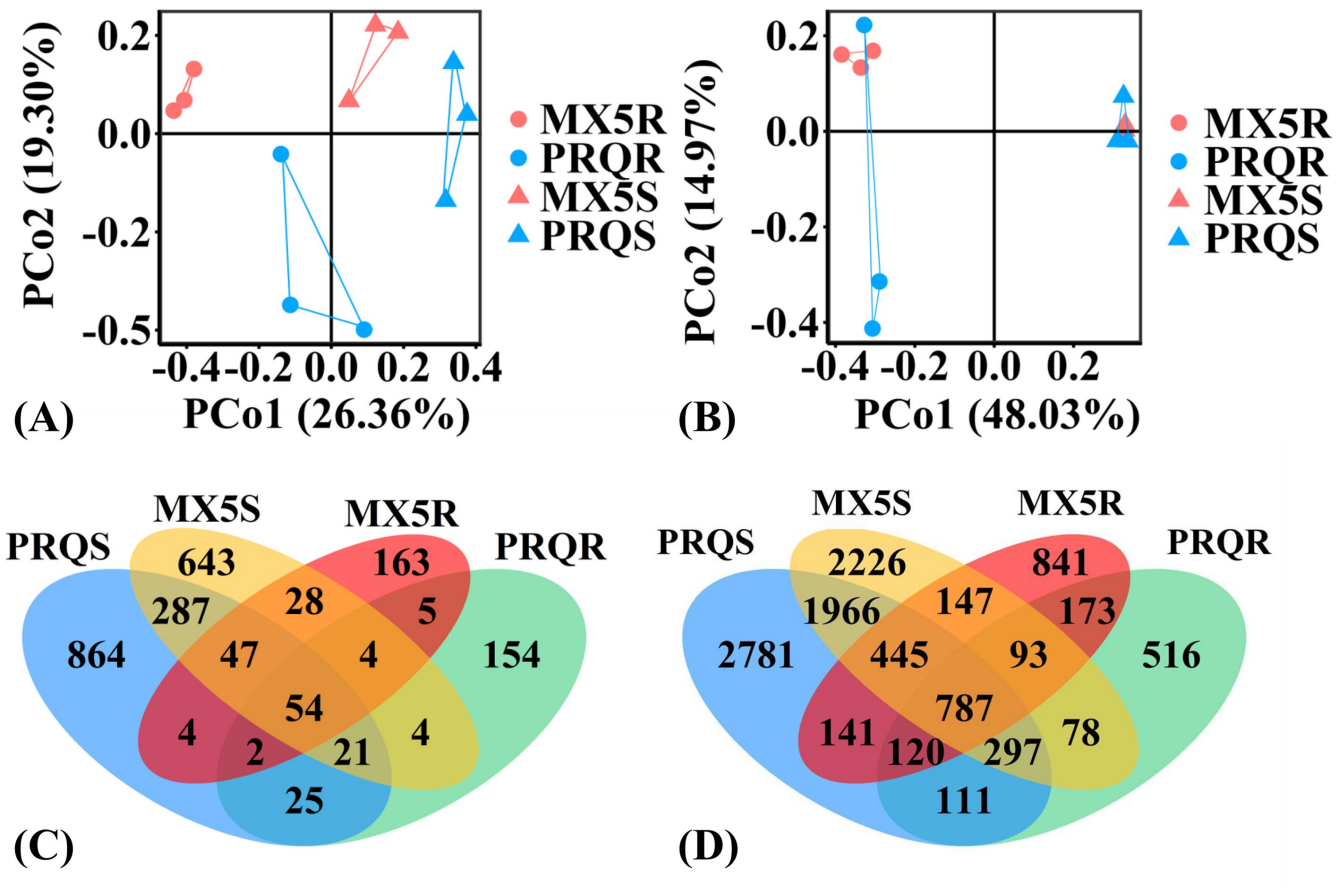
Principal coordinates analysis (PCoA) plots of the variation in the fungal **(A)** and bacterial **(B)** communities in the root (circle) and rhizosphere soil (triangle) of pecan grown with rootstocks 87MX5-1.7 (red) and Peruque (blue). Venn diagrams of different fungal **(C)** and bacterial **(D)** operational taxonomic units (OTUs) in pecan root and rhizosphere soil. The abbreviation is consistent with Figure 1.

The PCoA plots based on *β*-diversity illustrated the differences of fungal and bacterial communities among root/soil samples of two rootstocks (Figures 2A,B), which showed significance, as revealed by AMOVA results (*p* < 0.001). Furthermore, the compositions of fungal groups exhibited clear distinctions from each other (Figure 2A), while the compositions of bacterial groups varied between root and rhizosphere soil samples regardless of rootstock genotypes (Figure 2B). However, the area formed by the connection of bacterial groups in two rootstocks roots/soil intersect, suggesting that the effect of rootstock genotypes on bacterial groups was not as obvious compared to fungal groups.

In total, 54 fungal OTUs and 787 bacterial OTUs were discovered in both the roots and rhizosphere soil of the two rootstocks. The unique microbial communities present in different rootstocks were distinguishable from Venn diagrams (Figures 2C,D). Specifically, 87MX5-1.7 (MX5R and MX5S) exhibited 28 unique fungal OTUs and 147 unique bacterial OTUs in both root and soil, while Peruque (PRQS and PRQR) has 25 unique fungal OTUs and 111 unique bacterial OTUs (Figures 2C,D). The numbers of unique fungal and bacterial OTUs were higher in PRQS than in MX5S, while they were higher in MX5R than in PRQR.

### 3.3 Taxonomic composition of fungal communities in pecan rhizosphere soil and root with different rootstocks

**Figure 3:**
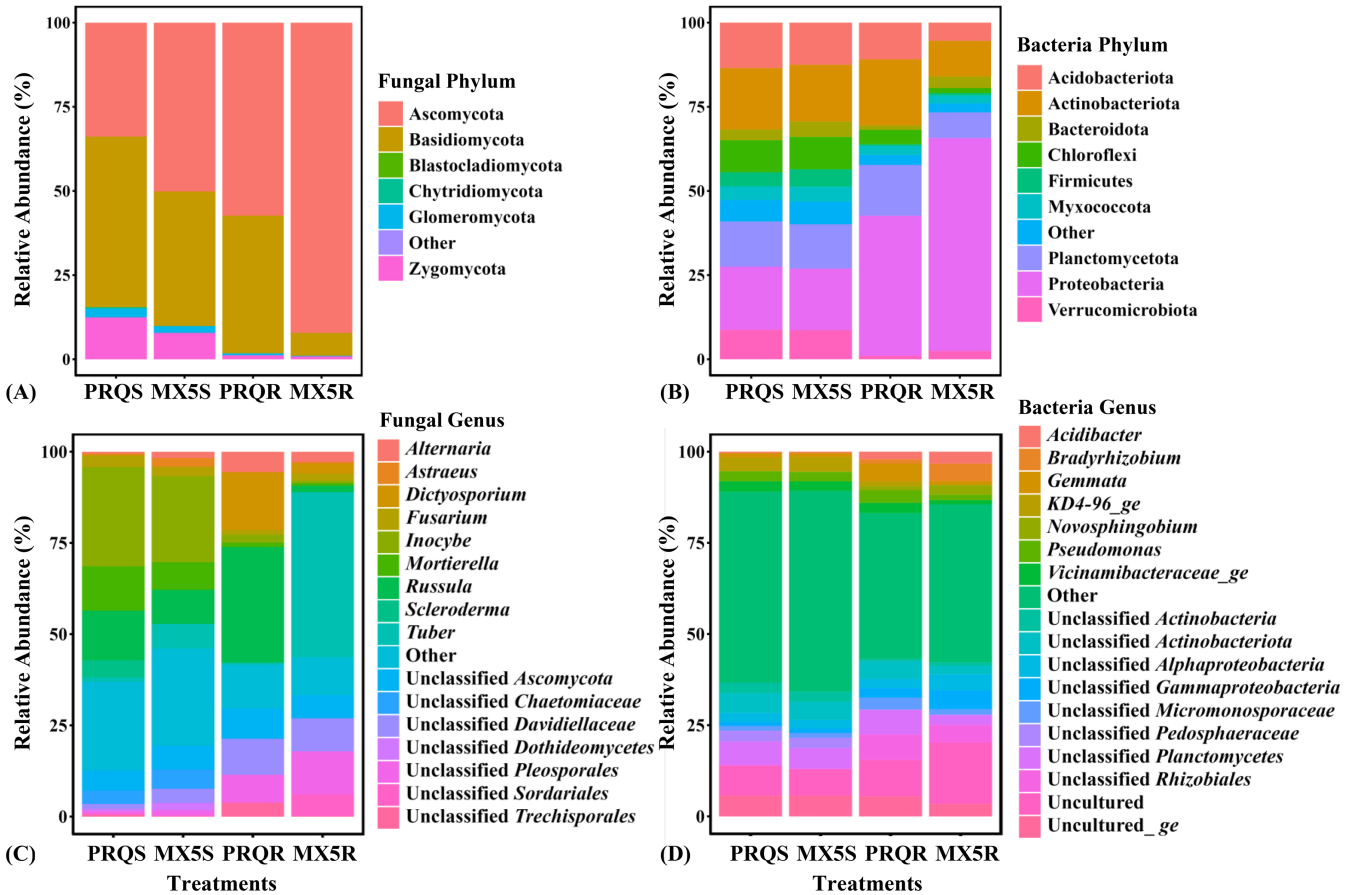
Relative abundance of fungal taxonomy at the phylum **(A)** and genus **(C)** levels, and bacterial taxonomy at the phylum **(B)** and genus **(D)** levels. Taxa with relative abundances below 0.05% at the fungal phylum level, 2% at the fungal genus level, 4% at the bacterial phylum level, and 2.5% at the bacterial genus level were categorized as “other”.

Among the 2,988 fungal OTUs from the roots and rhizosphere soil of the two rootstocks, Ascomycota prevailed as the predominant phylum in both the root and rhizosphere soil of 87MX5-1.7, and in the root of Peruque (Figure 3A). In contrast, Basidiomycota predominated in the rhizosphere soil of Peruque. Subsequently, Zygomycota and Glomeromycota showed notable proportions in the rhizosphere soil of the two rootstocks. However, the relative abundance of Glomeromycota showed considerably lower values in roots compared to that in rhizosphere soil (*P*_sample types_ = 0.015).

At class level (Supplementary Figure S1A), Agaricomycetes was the predominant fungal class in the rhizosphere soil of both rootstocks Peruque (49.64%) and 87MX5-1.7 (38.49%), as well as in the root of Peruque (40.25%). In contrast, Pezizomycetes was the predominant fungal class in the root of 87MX5-1.7, while Dothideomycetes exhibited a substantial presence at the root of Peruque.

For fungal orders (Supplementary Figure S1B), Agaricales (PRQS: 28.74%; MX5S: 24.41%), Russulales (PRQS: 13.62%; MX5S: 9.34%), and Mortierellales (PRQS: 12.14%; MX5S: 7.50%) were predominant in the rhizosphere soil of both Peruque and 87MX5-1.7. In the roots of Peruque, Russulales, Pleosporales, and Capnodiales ranked as the top three, with relative abundances of 31.65, 29.23, and 9.85%, respectively. For 87MX5-1.7, Pezizales (45.22%), Pleosporales (17.67%), and Capnodiales (9.04%) comprised the top three groups in the root fungal community.

In the context of fungal families (Supplementary Figure S1C), Inocybaceae (PRQS: 27.16%; MX5S: 23.58%), Russulaceae (PRQS: 13.62%; MX5S: 9.33%), and Mortierellaceae (PRQS: 12.13%; MX5S: 7.50%), were the top three in the rhizosphere soil of both Peruque and 87MX5-1.7. Notably, Tuberaceae predominated in the root of 87MX5-1.7 at 45.19%, while Russulaceae in the root of Peruque at 31.65%.

The fungal genera exhibited similarity in the rhizosphere soil of both Peruque and 87MX5-1.7 (Figure 3C), with predominant representatives being *Inocybe*, *Russula*, and *Mortierella*. *Tuber* was prevalent in the root of 87MX5-1.7 at 45.19%, while *Russula* was in the root of Peruque at 31.65%.

### 3.4 Taxonomic composition of bacterial communities in pecan rhizosphere soil and root with different rootstocks

A total of 10,722 bacterial OTUs were identified from the raw data of two rootstocks. There was no significant difference in the relative abundance of bacterial taxa in the rhizosphere soil of both Peruque and 87MX5-1.7 (Figures 3B,D). However, the composition of bacterial taxa in pecan roots varied among different treatments.

The predominant phyla observed in rhizosphere soil were Proteobacteria and Actinobacteriota (Figure 3B). In the root, Proteobacteria predominant in 87MX5-1.7, while Actinobacteriota predominant in Peruque.

At the class level, Gammaproteobacteria (PRQS: 10.07%; MX5S: 10.54%), Planctomycete (PRQS: 9.92%; MX5S: 9.31%), and Vicinamibacteria (PRQS: 8.66%; MX5S: 8.36%) were predominant in the rhizosphere soil (Supplementary Figure S1D). In the root, Alphaproteobacteria predominated in the root of 87MX5-1.7, while Actinobacteria and Vicinamibacteria predominated in the root of Peruque.

Regarding the order level (Supplementary Figure S1E), Vicinamibacterales (PRQS: 7.75%; MX5S: 7.39%) and unclassified Planctomycetes (PRQS: 6.41%; MX5S: 5.67%) were predominant in the rhizosphere soil. Rhizobiales was predominant in 87MX5-1.7 at 31.13%, whereas Vicinamibacterales in the root of Peruque at 7.49%.

The predominant bacterial families (Supplementary Figure S1F) in rhizosphere soil were unclassified Planctomycetes (PRQS: 6.41%; MX5S: 5.66%), unclassified Actinobacteriota (PRQS: 5.51%; MX5S: 5.00%), and Xanthobacteraceae (PRQS: 2.53%; MX5S: 1.77%). In the roots, Xanthobacteraceae (19.44%) and Sphingomonadaceae (6.45%) predominated in 87MX5-1.7, while Xanthobacteraceae (8.48%) and unclassified Rhizobiales (6.98%) predominated in Peruque.

At the genus level, unclassified Planctomycetes, unclassified Actinobacteriota were predominant in rhizosphere soil. In the roots, unclassified Planctomycetes and unclassified Rhizobiales predominated in Peruque, whereas *Bradyrhizobium*, unclassified Rhizobiales, and unclassified Gammaproteobacteria predominated in 87MX5-1.7 (Figure 3D).

### 3.5 The composition of fungal functional groups

The functional microbe composition, as defined using FUNGuild, exhibited differences between the two rootstock genotypes (Supplementary Figure S2). The saprotroph composition (Supplementary Figure S2A) was lower in root of 87MX5-1.7 at 3.83% compared to the Peruque root at 18.05% (*p* = 0.044). Meanwhile, the relative abundance of symbiotroph was higher in root of 87MX5-1.7 at 49.21% compared to in the root of Peruque at 35.31%, although this difference was not statistically significant (*p* = 0.767). In contrast, the saprotroph/symbiotroph group was significantly higher in the rhizosphere soil than in root (*p* = 0.034), with a higher presence in Peruque (PRQS: 12.51%) compared to 87MX5-1.7 (MX5S: 8.76%).

To accentuate the distinctions, the trophic modes from FUNGuild were merged into three categories: pathotroph (*p* = 0.339), saprotroph (*p* = 0.035), and symbiotroph (*p* = 0.132) in both pecan root and rhizosphere soil. The lowest relative abundance of saprotroph was observed in the 87MX5-1.7 root at 13.05% (Supplementary Figure S2D). In the Peruque root, the relative abundance of symbiotrophs (PRQR: 45.69%; MX5R: 57.49%) was lower, while that of pathotrophs (PRQR: 14.73%; MX5R: 11.44%) was higher compared to 87MX5-1.7 (Supplementary Figures S2C,E).

Further scrutiny of FUNGuild’s ecological guilds revealed that ectomycorrhizal fungi were the pivotal taxa in both pecan roots and rhizosphere soil, with an averaging relative abundance of 43.37%, regardless of the rootstock genotype (Supplementary Figure S2B). In the Peruque root, the relative abundance of ectomycorrhizal fungi was the lowest at 34.72%, while the relative abundances of plant saprotroph/undefined saprotroph (*p* = 0.087), animal pathogen/dung saprotroph/endophyte/epiphyte/plant saprotroph/wood saprotroph (*p* = 0.034), animal pathogen/endophyte/plant pathogen/plant saprotroph/wood saprotroph (*p* = 0.236) and plant pathogen (*p* = 0.386) were higher than in the root of 87MX5-1.7. Similarly, the relative abundance of endophyte/plant saprotroph/undefined saprotroph was higher in the rhizosphere soil of Peruque than in 87MX5-1.7 (*p* = 0.057). However, the relative abundances of ectomycorrhizal fungi (PRQS: 47.58%; MX5S: 42.15%; *p* = 0.792) and arbuscular mycorrhizal fungi (PRQS: 1.56%; MX5S: 0.97%; *p* = 0.111) were higher in the rhizosphere soil of Peruque than in 87MX5-1.7.

To investigate unique fungal groups in different rootstock genotypes, the overlapping fungal OTUs from Venn diagram in Figure 2C were extracted and annotated based on their ecological guilds (Supplementary Figure S3). The overlap between the two rootstocks (including both root and rhizosphere soil samples) primarily consisted of 13.0% plant pathogens, 11.1% ectomycorrhizal fungi, and 5.6% undefined saprotrophs (Supplementary Figure S3A). The unique fungal groups in the root and rhizosphere soil of 87MX5-1.7 predominantly comprised 14.3% ectomycorrhizal fungi and 3.6% arbuscular mycorrhizal fungi (Supplementary Figure S3B). Similarly, the unique fungal communities of Peruque were largely composed of 28.0% ectomycorrhizal fungi, 12.0% arbuscular mycorrhizal fungi, and 8.0% undefined saprotrophs (Supplementary Figure S3C).

Fungal genera with a sum of relative abundances exceeding 1% among treatments associated with ectomycorrhizal fungi, arbuscular mycorrhizal fungi, and plant pathogens, were extracted and listed in Table 1. Arbuscular mycorrhizal fungi, including *Glomus macrocarpum*, unclassified *Rhizophagus*, unclassified Glomeraceae, and unclassified Glomeromycota, were detected in the rhizosphere soil but were scarcely present in the roots. Ectomycorrhizal fungi included *Astraeus*, *Hebeloma*, *Helvella*, *Inocybe*, *Russula*, *Scleroderma*, unclassified Sebacinaceae, and *Tuber*. Notably, the relative abundance of *Tuber* was significantly higher in the root of 87MX5-1.7 compared to Peruque (*p* < 0.001), with rootstock genotype, sample type, and their interaction effects all contributing to these variances, as indicated by between-subjects effect analyses (Table 2). Furthermore, the relative abundances of *Russula*, *Scleroderma*, unclassified *Sebacinaceae* were higher in PRQR than in MX5R, although not statistically significant. Similarly, the relative abundance of *Inocybe* was higher in the rhizosphere soil than in the root, although not statistically significant.

**Table 1:** The relative abundance of fungal genus in pecan root and rhizosphere soil.

Genus	Category	MX5R (%)	PRQR (%)	MX5S (%)	PRQS (%)	*F*	*P*
Unclassified Glomeraceae	AMF	0.078 ± 0.056	0.034 ± 0.034	0.443 ± 0.205	0.999 ± 0.406	3.782	0.059
Unclassified Glomeromycota	AMF	0.041 ± 0.036	0 ± 0	0.25 ± 0.16	0.129 ± 0.115	1.219	0.364
*Glomus macrocarpum*	AMF	0 ± 0	0.004 ± 0.004	0.284 ± 0.142	0.278 ± 0.248	1.272	0.348
Unclassified *Glomus*	AMF	0 ± 0	0 ± 0	0.254 ± 0.127	0.27 ± 0.142	2.526	0.131
Unclassified *Rhizophagus*	AMF	0 ± 0	0.286 ± 0.144	0 ± 0	0.045 ± 0.043	3.310	0.078
*Astraeus*	ECMF	0.45 ± 0.271	0.024 ± 0.019	2.382 ± 1.276	0.239 ± 0.113	2.752	0.112
*Hebeloma*	ECMF	0 ± 0	0.034 ± 0.034	0 ± 0	0.417 ± 0.403	1.011	0.437
*Helvella*	ECMF	0.016 ± 0.016	0.063 ± 0.052	0.035 ± 0.035	0.295 ± 0.286	0.789	0.533
*Inocybe*	ECMF	0.613 ± 0.562	2.185 ± 0.836	23.579 ± 13.792	27.162 ± 13.608	2.063	0.184
*Russula*	ECMF	1.783 ± 1.74	31.652 ± 11.183	9.327 ± 9.279	13.621 ± 3.609	2.833	0.106
*Scleroderma*	ECMF	0 ± 0	0.178 ± 0.145	0 ± 0	4.697 ± 4.694	0.977	0.450
Unclassified *Sebacinaceae*	ECMF	0.751 ± 0.711	0.126 ± 0.097	0 ± 0	0.009 ± 0.009	0.994	0.443
*Tuber*	ECMF	45.195 ± 8.775a	0.46 ± 0.322b	6.752 ± 2.8b	1.133 ± 1.12b	21.240	**0.000**
*Cylindrocarpon*	Plant PAT	0.436 ± 0.278	0.094 ± 0.094	0.246 ± 0.063	0.045 ± 0.027	1.365	0.321
*Ilyonectria*	Plant PAT	0.946 ± 0.231a	0.25 ± 0.22b	0.09 ± 0.027b	0.147 ± 0.013b	6.145	**0.018**
*Lectera*	Plant PAT	0.067 ± 0.06b	0.023 ± 0.023b	0.711 ± 0.294a	0.083 ± 0.054b	4.603	**0.037**
*Monographella*	Plant PAT	1.173 ± 0.46	0.744 ± 0.499	1.537 ± 0.62	0.322 ± 0.162	1.273	0.348
*Neonectria*	Plant PAT	0.229 ± 0.126	1.446 ± 1.273	0.456 ± 0.236	0.368 ± 0.102	0.725	0.565
*Pestalotiopsis*	Plant PAT	0.241 ± 0.125	0.465 ± 0.425	0.017 ± 0.01	0.003 ± 0.003	0.969	0.454

The data were shown as means ± standard error (*N* = 3). Genera with total relative abundances in both root and rhizosphere soil of the selected two rootstocks above 0.01% were selected. Different lowercase letters indicated significant differences among the means by Duncan’s test (*P* < 0.05). The *p*-values lower than 0.05 were bolded to highlight. “ND” represents that this genus was not detected in the current treatment. AMF, arbuscular mycorrhizal fungi, ECMF, ectomycorrhizal fungi; PAT, pathotroph. The other abbreviation was consistent with Figure 1.

**Table 2:** Tests of between-subjects effects among unique fungal genera.

Genus		Sample type	Genotypes	Sample type × Genotypes
*Tuber*	*F*	16.32	28.964	17.504
	*P*	**0.004**	**<0.001**	**0.003**
*Ilyonectria*	*F*	8.909	3.948	5.495
	*P*	0.170	0.820	0.470
*Lectera*	*F*	5.297	4.834	3.639
	*P*	**0.050**	0.059	0.093

The *p*-values no more than 0.05 were bolded to highlight.

The category of plant pathogens comprised *Cylindrocarpon*, *Ilyonectria*, *Lectera*, *Monographella*, *Neonectria*, and *Pestalotiopsis*. *Ilyonectria* showed a significantly higher (*p* = 0.018) abundance in the root of 87MX5-1.7 (0.943%) compared to Peruque (0.250%), while *Lectera* showed a significantly higher (*p* = 0.037) abundance in the rhizosphere soil of 87MX5-1.7 (0.710%) compared to Peruque (0.083%). Between-subjects effect analyses (Table 2) indicated that rootstock genotype contributed to this difference in *Lectera* abundance. Additionally, *Monographella* presented the highest relative abundance in the root of 87MX5-1.7 (1.169%), while *Neonectria* presented the highest relative abundance in the root of Peruque (1.446%).

### 3.6 The composition of bacterial functional groups

Functional bacterial groups were defined using FAPROTAX (Supplementary Figure S4), with a specific emphasis on accentuating the functional groups that exhibited major distinctions between the two selected rootstock genotypes. The abundance of nitrogen fixation related groups in roots was higher in 87MX5-1.7 than those in Peruque (*p* = 0.059). Similarly, groups related to nitrate reduction (*p* = 0.052) in both roots and rhizosphere soil were present in higher amounts in 87MX5-1.7 compared to those with Peruque. Additionally, the abundance of fermentation-related groups in rhizosphere soil was higher in 87MX5-1.7 compared to those with Peruque (*p* = 0.274).

To delve deeper into the distinct contributions of microbial communities to nitrogen uptake in pecans with the two rootstock genotypes, bacterial genera related to nitrogen fixation and nitrate reduction with relative abundances above 0.01% were examined and summarized in Table 3. *Bradyrhizobium*, associated with nitrogen fixation, exhibited significantly higher abundance (*p* = 0.044) in the root of 87MX5-1.7 (4.768%) compared to Peruque (1.169%), but showed no differences in the rhizosphere soil of two rootstocks. Between-subjects effect analyses (Table 4) indicated that sample type significantly influenced the relative abundance of *Bradyrhizobium*. Nitrate reduction included *Achromobacter*, *Aeromonas*, *Aquabacterium*, *Brucella*, *Emticicia*, *Ensifer*, *Rubrobacter*, and *Stenotrophomonas*. Of them, *Achromobacter* was more abundant in the root of 87MX5-1.7 (0.204%) than in Peruque (0.002%). *Ensifer* had higher relative abundance in the root of 87MX5-1.7 (0.188%) than that in Peruque (0.086%), while it was slightly higher in the rhizosphere soil of Peruque (0.190%) than in 87MX5-1.7 (0.160%). Most bacterial groups showed higher relative abundances in rhizosphere soil rather than in roots. However, only *Aquabacterium* (*p* = 0.045) showed significantly higher relative abundances in the rhizosphere soil of 87MX5-1.7 than in Peruque, whereas *Rubrobacter* exhibited higher relative abundances in the rhizosphere soil of Peruque compared to 87MX5-1.7. Between-subjects effect analyses (Table 4) indicated that the significant contribution of sample type to the relative abundance of *Aquabacterium*.

**Table 3:** The relative abundance of bacterial genus in pecan root and rhizosphere soil.

Genus	Category	MX5R (%)	PRQR (%)	MX5S (%)	PRQS (%)	*F*	*P*
*Bradyrhizobium*	Nitrogen fixation	4.768 ± 1.906a	1.169 ± 0.818b	0.245 ± 0.043b	0.240 ± 0.079b	4.305	**0.044**
*Achromobacter*	Nitrate reduction	0.204 ± 0.200	0.002 ± 0.002	ND	0.001 ± 0.001	1.027	0.431
*Aeromonas*	Nitrate reduction	0.002 ± 0.002	0.004 ± 0.004	0.708 ± 0.687	0.017 ± 0.014	1.040	0.426
*Aquabacterium*	Nitrate reduction	ND	ND	0.010 ± 0.005a	0.007 ± 0.002ab	4.248	**0.045**
*Brucella*	Nitrate reduction	ND	0.005 ± 0.005	0.004 ± 0.004	0.002 ± 0.002	0.423	0.742
*Emticicia*	Nitrate reduction	0.007 ± 0.007	ND	ND	ND	1.000	0.441
*Ensifer*	Nitrate reduction	0.188 ± 0.071	0.086 ± 0.031	0.160 ± 0.009	0.196 ± 0.045	1.249	0.355
*Rubrobacter*	Nitrate reduction	0.003 ± 0.003	ND	0.089 ± 0.039	0.143 ± 0.076	2.701	0.116
*Stenotrophomonas*	Nitrate reduction	0.008 ± 0.001	ND	0.004 ± 0.002	0.004 ± 0.004	2.001	0.192

The data were shown as means ± standard error (*N* = 3). Genera with total relative abundances in both root and rhizosphere soil of the selected two rootstocks above 0.01% were selected. “ND” represents that this genus was not detected in the current treatment. Different lowercase letters indicated significant differences among the means by Duncan’s test (*p* < 0.05). The *p*-values no more than 0.05 were bolded to highlight. The abbreviation was consistent with Figure 1.

**Table 4:** Tests of between-subjects effects among unique bacterial genera.

Genus		Sample type	Genotypes	Sample type × Genotypes
*Bradyrhizobium*	*F*	6.902	3.014	3.000
	*P*	**0.030**	0.121	0.122
*Aquabacterium*	*F*	11.901	0.422	0.422
	*P*	**0.009**	0.534	0.534

The *p*-values no more than 0.05 were bolded to highlight.

## 4 Discussion

**Figure 4:**
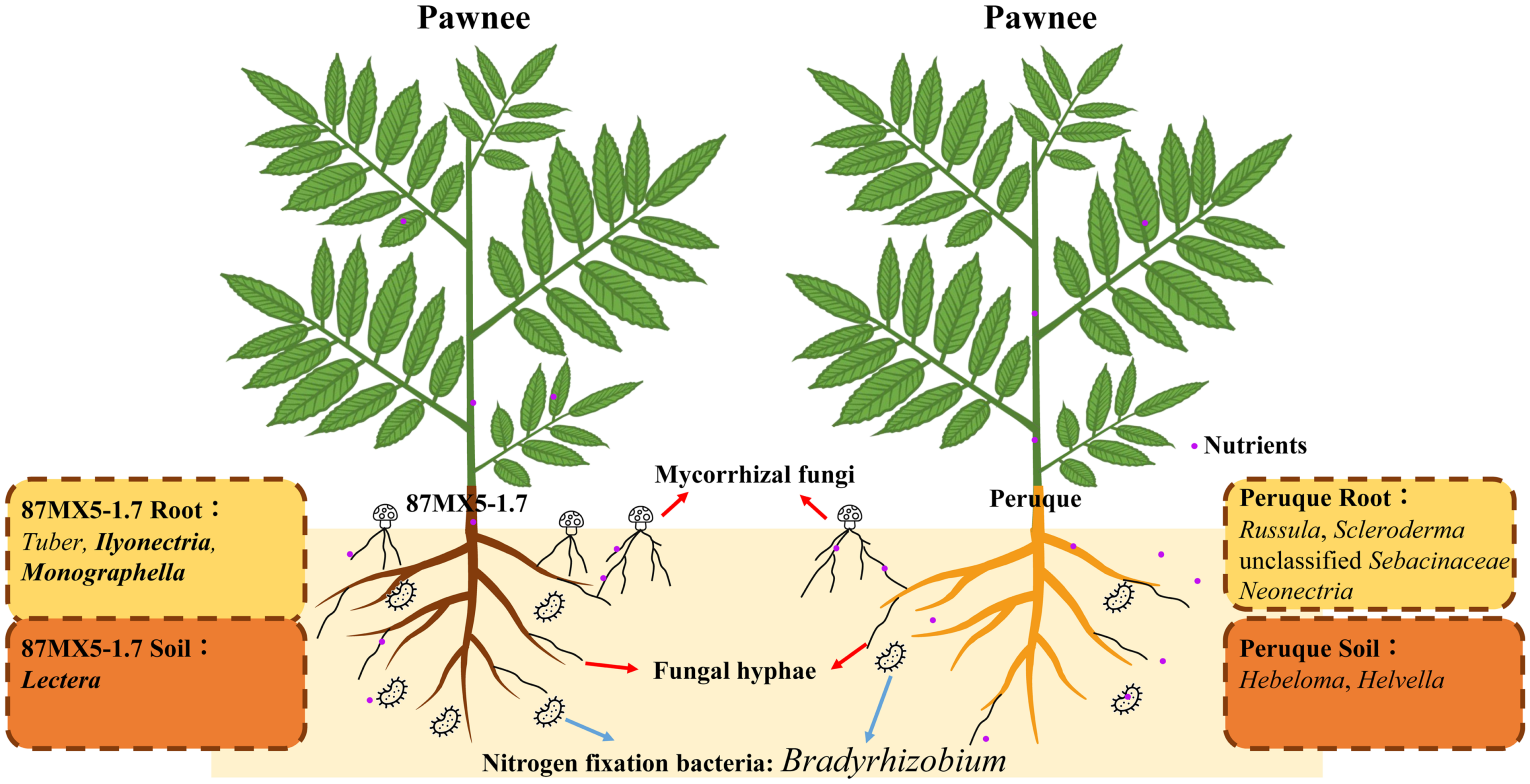
Schematic representation of the pecan-soil interactions and experimental design.

Rootstock refers to the lower part of grafted combinations, which can affect plant growth, scion performance, and nut quality (Fallahi et al., 2001). The performance of rootstocks is subject to its genotype and soil environments. Previous studies have observed that rootstock genotypes and their root exudates play a crucial role in influencing microbial diversity and metabolism in the rhizosphere soil, which may consequently impact fertilizer availability and the suppression of diseases (Dries et al., 2021; Marasco et al., 2018). In this study, two rootstock genotypes from northern (Peruque from Peruque, St. Charles, MO, United States) and southern (87MX5-1.7 from Jaumave, Tamaulipas, Mexico) regions of north America were selected, as depicted in Figure 4, to elucidate the interactions among pecan rootstock, soils, and associated microbial communities. The unique fungal symbiotroph and pathotroph groups (highlighted in bold), as well as nitrogen-fixing bacteria, in the roots/soil of the two rootstocks were specifically focused on.

### 4.1 The rootstock genotypes shaped microbial communities

Compared to scion, the roots of rootstocks directly contact with soil to absorb nutrients, potentially having a greater impact on plant’s nutrient absorption and rhizosphere microbial communities (Castellano-Hinojosa et al., 2023). Numerous studies have shown that plant genotypes greatly affect the rhizosphere microbial communities (Mendes et al., 2013; Xun et al., 2024). In this study, samples collected from the same orchard revealed no significant differences in soil characteristics were observed between the two rootstocks (Supplementary Table S2). However, statistically significant differences were observed in The p-values no more than 0.05 were bolded to highlight. the microbial communities (*α*-diversity indices) between the roots and rhizosphere soil of two rootstocks (Figure 1). The results of PCoA (Figures 2A,B), based on *β*-diversity, indicated that fungal communities varied across different treatments, whereas bacterial communities differed based on sample types (root or rhizosphere soil). This highlights the influence of pecan rootstock genotypes on fungal communities. Fungal communities were found to be more sensitive to pecan genotypes than bacterial communities (Liu et al., 2022a). Similar results have also been observed on apples that fungal communities were highly affected by plant genotypes and were more influenced than bacterial communities (Liu et al., 2022b). The host pecan’s preference for fungal communities appeared more restricted than for bacterial communities, probably because the host pecan heavily relied on nutrients delivered by its fungal partner, such as mycorrhizal fungi. The research of legumes has shown that the reproductive success of rhizobia was reduced when rhizobia struggle to provide adequate nitrogen for host plant growth and photosynthesis (Kiers et al., 2003). In this way, the nutrient and/or water requirement from the host plant may be the primary reason for adjusting and attracting the microbial partner.

The mutual interaction between host plants and their microbial partners can be facilitated by various plant metabolites (i.e., root exudates), and different genotypes of host plants with varying metabolites could attract different microbes to form symbiosis or cause disease (Berg et al., 2014; Mendes et al., 2013; Spence and Bais, 2015). The distinct differences observed in fungal communities, characterized by higher fungal richness (Figure 1E) and more OTUs (Figure 2C) in rhizosphere soil than in roots, suggested that not all fungi present in the rhizosphere soil can infect pecans root. The relative abundance of *Inocybe* was higher in the rhizosphere soil than in the root (although not statistically significant) regardless of rootstock genotypes (Table 2). This further indicated that pecans exhibit a preference in selecting fungal partners to form symbiotic relationships, while other soil fungi may play supportive roles in adjusting the rhizosphere microenvironment. Although diversity indices did not show statistical differences between the two rootstocks (Figure 1), the compositions of microbes and microbial function groups were notably different between the two rootstocks (Figure 2 and Supplementary Figures S1–S4). We observed 25 fungal OTUs unique to Peruque and 28 fungal OTUs unique to 87MX5-1.7. The identification of unique OTUs in the microbial communities associated with the two rootstocks suggested a potential influence of the rootstock genotype on the composition of the root and rhizosphere microbiomes. These unique OTUs could have significant implications for the health and growth of pecan trees, potentially contributing to differences in nutrient uptake, disease resistance, or stress tolerance between the rootstocks.

There were variations in the relative abundances of symbiotrophs, saprotrophs, and pathotrophs between the two rootstocks (Supplementary Figure S2), indicating that the functionality of fungal communities was influenced by the genotypes of the rootstock. Specifically, the relative abundance of symbiotrophs was lower while the relative abundance of saprotrophs was higher in the roots of Peruque, compared to those in the roots of 87MX5-1.7 (Supplementary Figure S2A), suggesting a different trophic mode between the two rootstock genotypes. ECM fungi and AM fungi were predominantly found in the unique groups associated with 87MX5-1.7 and Peruque (Supplementary Figures S3B,C), suggesting that these mycorrhizal fungi have a higher preference for pecan hosts. The slightly differences in the relative abundances of these related mycorrhizal fungi between the two genotypes may be attributed to variations in root exudates from different genotypes of rootstock.

In this study, the bacterial communities appeared to be unaffected by rootstock genotypes but were affected by sample type, indicating a relatively stable and different bacterial community in pecan roots and rhizosphere soil separately. Marasco et al. (2018) have observed that grapevine rootstock affected bacterial communities but did not impact their potential functionality. Continuous studies have revealed an increasing number of cooperative associations between bacteria and fungi in rhizosphere (Bonfante and Desiro, 2017; de Novais et al., 2020). For example, *Rhizophagus intraradices*, an AM fungus, has been found to transfer carbon from *Avena barbata* to surrounding bacteria belonging to the phyla Myxococcota, Fibrobacterota, Verrucomicrobiota, as well as the ammonia-oxidizing archaeon genus *Nitrososphaera* (Nuccio et al., 2022). Generally, AM fungi provide nutrients to host plants in exchange for carbon, facilitating the survival of both the fungus and the plant (Kiers et al., 2011). The bacterial communities may contribute as additional nutrient supporters by promoting nutrient release from the soil and collaborating with rhizosphere fungi to provide nutrients to the host plant. As a result, the changes in bacterial communities were driven more by environmental factors rather than the genotypes of the rootstock, consistent with what observed in the current study.

### 4.2 Rootstock attracted different fungal symbiotroph and pathotroph groups

Symbiotroph is a group of microbes capable of forming symbiotic relationships with host plants, playing a crucial role in influencing nutrient transportation within the plant, promoting plant growth, and protecting plants from pathogen infections (Li et al., 2021; Smith and Smith, 2011). This group was the most abundant in both pecan roots and rhizosphere soil of both rootstocks (Supplementary Figure S2A). Mycorrhizal fungi, including both ECM fungi and AM fungi, have been predominantly observed in pecan rhizosphere in previous studies (Liu et al., 2022a; Ren et al., 2023). These fungi play a crucial role in nutrient acquisition, ultimately enhancing pecan growth and improving nut quality (Babuin et al., 2016; Flores-Cordova et al., 2018).

In this study, detailed annotation revealed a high abundance of ECM fungi in the pecan microbial communities (Supplementary Figure S2B). The composition of ECM fungi belongs to the genera of *Astraeus*, *Hebeloma*, *Helvella*, *Inocybe*, *Russula*, *Scleroderma*, unclassified Sebacinaceae, and *Tuber* (Table 2). The presence of *Tuber*, *Astraeus*, *Russula*, *Scleroderma*, and *Inocybe* in pecans has been documented in previous studies (Bonito et al., 2011; Freiberg et al., 2021; Ge et al., 2017). However, the existence of *Hebeloma*, *Helvella* and unclassified Sebacinaceae have been rarely observed. The unclassified Sebacinaceae were noted in the roots of both rootstocks 87MX5-1.7 and Peruque, with relative abundances of 0.751% (MX5R) and 0.126% (PRQR), respectively, suggesting that it may play a specific role in the pecan lifecycle. However, due to its low abundance and unclassified status at the genus level, its exact role remains unclear and may indicate a minor role in the pecan lifecycle, warranting further analysis to determine its significance. The relative abundance of *Hebeloma* was not detected in 87MX5-1.7, while it comprised only 0.417% in the rhizosphere soil and 0.03% in the root of Peruque (Table 2). The relative abundances of *Helvella* were less than 0.05% in the roots and rhizosphere soil of 87MX5-1.7, while they comprised low abundances (PRQR: 0.063%; PRQS: 0.295%) Peruque. These results suggested that Peruque formed a stronger relationship with *Hebeloma* and *Helvella* compared to 87MX5-1.7.

Most ECM fungi were different between the two rootstock genotypes, with *Tuber* being predominant in 87MX5-1.7 roots and *Russula* and *Scleroderma* being predominant in the Peruque roots (Table 2). The most well-known *Tuber* in pecans, named pecan truffle, is an edible fungus that can improve pecan growth (Hamilton, 2014; Hanlin et al., 1989). The symbiosis between pecans and *Tuber* is considered valuable for providing additional benefits such as producing truffle as byproducts or improving pecan growth conditions to advanced pecan orchard management (Bonito et al., 2011), the variations in the relative abundance of *Tuber* between different rootstocks may affect truffle production and attention is needed for the successful establishment of a truffle-pecan co-farming orchard.

Arbuscular mycorrhizal (AM) fungi, including *Glomus macrocarpum*, unclassified *Glomus*, unclassified *Rhizophagus*, unclassified Glomeraceae, and unclassified Glomeromycota, were found in the rhizosphere soil but not in the roots (Table 1). Although their relative abundance in the rhizosphere soil was readable, their presence in the endospheric root was too low to detect. With unclassified *Rhizophagus* detected at only 0.286% relative abundance in the root of Peruque, it suggested that AM fungi may play a minor role in pecan symbiosis with variations across different rootstock genotypes. This differs from the findings in an experimental pecan orchard in Oklahoma (Ren et al., 2023), where AM fungi were found in the roots of pecan trees from these two selected rootstocks. This suggests that the relationship between pecans and AM fungi varies not only among different pecan genotypes but also among different rootstocks. This result also sheds light on pecan’s selection of AM fungi and ECM fungi. While 87MX5-1.7 showed a stronger symbiotic relationship with ECM fungi *Tuber*, Peruque demonstrated a preference to form symbiosis with ECM fungi *Russula* (31.652%) and *Inocybe* (2.185%), as well as AM fungi unclassified *Rhizophagus* (0.286%). Our results suggested that Peruque was in favor of multiple fungi to gain benefits, while 87MX5-1.7 preferred a specific domain of fungi to form beneficial symbiosis.

Among the major plant pathotroph fungi including *Cylindrocarpon*, *Ilyonectria*, *Lectera*, *Monographella*, *Neonectria*, and *Pestalotiopsis*, *Monographella* exhibited the highest relative abundance in both the root and rhizosphere soil of 87MX5-1.7, *Neonectria* showed the highest relative abundance in both the root and rhizosphere soil of Peruque. A previous study has observed an increased abundance of *Monographella* when *Tuber borchii* was inoculated to *Corylus avellana* (Li et al., 2019), indicating a potential preferred selective relationship between *Monographella* and *Tuber*. Certain members of *Monographella* can induce fusarium patch and snow molds on crops/grasses (Perry, 1986; Shu and Xiong, 1993) although their pathogenicity to pecans is not extensively documented. Additionally, *Monographella cucumerina* and *Lectera longa* have been detected in other pecan orchards (Ren et al., 2023), suggesting their common presence in such environments. Some members of *Ilyonectria* can cause root rot in trees and many crops (Farh et al., 2018; Manici et al., 2018), while the members of *Lectera* can cause leaf spot on crops (Cannon et al., 2012; Eken et al., 2002). Members of the genus *Neonectria* are known to cause canker and black foot disease on fruit trees and other woody plants (Halleen et al., 2006; Weber and Børve, 2021). The variation in pathotroph fungi indicated a potential risk of Fusarium patch and snow molds in pecan orchards with 87MX5-1.7 as a rootstock, while canker and black foot disease should be paid special attention in pecan orchards with Peruque as a rootstock.

### 4.3 The genotype of the rootstock, characterized by its abundance of nitrogen-fixing bacteria and symbiotroph fungi, may impact pecan growth

Bacteria are essential components of the plant rhizosphere, contributing significantly to environmental structure and nutrient cycling for plants (Kuypers et al., 2018). Analysis of functional bacterial groups revealed differences in groups associated with nitrogen fixation and nitrate reduction between two rootstocks (Figure 4). Previous research has highlighted the substantial contributions of nitrogen-related bacteria in pecan orchards subjected to different levels of nitrogen fertilization (Ren et al., 2023). Notably, nitrogen-related bacteria were found to be influenced by the genotypes of the rootstock in this study. Specifically, the relative abundance of *Bradyrhizobium*, a well-known nitrogen-fixation bacteria, was significantly higher in pecan roots of 87MX5-1.7 compared to those of Peruque (Table 3). *Bradyrhizobia* and rhizobia form a symbiotic relationship, facilitating the formation of nitrogen-fixing nodules on legumes (Jaiswal and Dakora, 2019; Kvien and Pallas, 2008; Parker and Kennedy, 2006). The research on radishes (*Raphanus sativus*) (Antoun et al., 1998) have confirmed the symbiosis with ability to enhance the growth in non-legumes. Although limited literature exists on the direct enhancement of pecan growth by *Bradyrhizobia*, its presence has been observed in pecan rhizosphere soil with high relative abundance (Shi et al., 2023).

The different genotypes of rootstock resulted in different heights and diameters of pecans under similar soil conditions (Supplementary Table S1), suggesting distinct nutrient acquisition strategies. Peruque with a higher abundance of saprotroph fungi suggested that pecans may obtain nutrients from the decomposition of organic matter by these fungi. As litter constitutes the primary source of saprotroph and undergoes seasonal changes (Fu et al., 2017), it indicated a potential unstable nutrient supply for Peruque. Consequently, pecans cultivated with Peruque need multiple fungi to gain enough benefits. On the contrary, the high abundance of symbiotroph fungi and nitrogen-fixing bacteria in 87MX5-1.7, suggested a strategy focused on high nutrient input. Despite a higher abundance of pathogen in the rhizosphere soil (Supplementary Figure S2), 87MX5-1.7 showed greater height and diameter compared to those with Peruque (Supplementary Table S1). The substantial high abundance of ECM fungi in the rhizosphere soil of 87MX5-1.7 suggested their ability to survive pathogenic environments. This resilience could be attributed to the ecological trait of ECM fungi shielding against pathogen infection. By forming a dense network of hyphae around the root tips and producing secondary metabolites, ECM fungi acted as a barrier to prevent pathogens from entering the root system and promoting plant growth (Dyshko et al., 2024). Also, the rhizosphere bacterial communities could contribute to the plant disease resistance (Carrion et al., 2019). In summary, the rootstock genotype significantly impacted the nutrient acquisition strategy and defense mechanisms against pathogens of host plants, thus leading to varied growth conditions.

### 4.4 Core microbiome

The core microbiome refers to a group of microbes consistently found in a specific host or environment (Neu et al., 2021). Cervantes et al. (2022b) demonstrated that the core microbiome can transfer from pecan seeds to seedlings. This suggested that the core microbiome plays a crucial role in pecan growth and may have significant effects on seed germination and seedling development. By analyzing the core microbiome of pecans, future research can delve deeper into the roles and influences of microbes in pecan processes. Previous research observed a core microbiome among pecan cultivars Burkett, Mandan, Pawnee, Western, and Wichita but did not provide functional annotation (Cervantes et al., 2022a). In our study, the ECM fungi and plant pathogens predominated (Supplementary Figure S3A) among these shared fungal OTUs (Figure 2 and Supplementary Figure S3A), indicating the presence of a core microbiome within these two selected pecan rootstocks, primarily composed of ECM fungi and plant pathogens.

Among pecan cultivars Burkett, Mandan, Pawnee, Western, and Wichita, core microbiome families identified among in pecan leaves, stems, and roots included Serendipitaceae, Nectriaceae, Ophiostomataceae, Hypocreaceae, Aspergillaceae, and Cephalothecaceae of pecans planted in New Mexico (Cervantes et al., 2022a). In this study, core microbiome families of pecan root and rhizosphere soil with high total relative abundance (>50%) included Russulaceae, Tuberaceae, and Inocybaceae. After merging the low relative abundance group as “other,” the core microbiome families observed in this study differed from those in New Mexico orchard (Cervantes et al., 2022a). The differences observed in core microbiome families between our study and the previous research conducted in the New Mexico pecan orchard (Cervantes et al., 2022a) may stem from a combination of factors, including variations in rootstock genotypes and geographical locations. Results of this study indicated a core microbiome comprising the ECM fungal genera of *Astraeus*, *Helvella*, *Inocybe*, *Russula*, unclassified Sebacinaceae, and *Tuber*. Among them, *Tuber*, *Astraeus*, and *Russula*, are ECM fungi commonly found in pecans (Bonito et al., 2011; Ge et al., 2017), indicating their important role in pecan growth and highlighting the key role of ECM fungi in pecan lifecycle. As for pathogens, the core microbiome in this study included genera of *Cylindrocarpon*, *Gibberella*, *Ilyonectria*, *Lectera*, *Monographella*, *Neonectria*, and *Pestalotiopsis*. These pathogens may coexist with pecan trees throughout their lifespan, but only become a significant issue when they break out at specific condition (Abdullah et al., 2017; Velasquez et al., 2018). Our study annotated the detailed information in core microbiome, with specific beneficial ECM fungi and pathogen. Monitoring changes in the core microbiome of pecan rhizosphere soil can help better predict the risk of pecan disease, thereby providing suitable guidance for pecan orchard managements.

In this study, a total of 787 bacterial OTUs were found to be shared in the roots and rhizosphere soil of both rootstock genotypes (Figure 2). Within this extensive bacterial community, Xanthobacteraceae, Sphingomonadaceae, unclassified Planctomycetes, unclassified Actinobacteriota, unclassified Rhizobiales, and unclassified Gammaproteobacteria comprised the core microbiome at the family level. Our findings, combining the results of bacterial core microbiome identified in the New Mexico orchard (Cervantes et al., 2022a), which included Rhizobiaceae, Enterobacteriaceae, Chitinophagaceae, Burkholderiaceae, Sphingomonadaceae, Pseudomonadaceae, Moraxellaceae, Microscillaceae, Rubritaleaceae, and Caulobacteraceae, revealed that Sphingomonadaceae is the bacterial core microbiome of pecans. This suggested that although the rhizosphere microbiomes were mostly influenced by rootstock genotype and geographical locations, certain components of the microbiomes form stable core microbiomes within the pecan rhizosphere. Future research should prioritize expanding the core microbiome database by investigating various seasons, genotypes, and locations to provide a comprehensive understanding of pecan microbiomes.

## 5 Conclusion

In this study, different rootstocks exhibited variations in growth and microbial communities, despite being cultivated under similar soil conditions (Figure 4). These variations were likely attributed to the different pathways of nutrient translocation in pecans, influenced by the composition of nitrogen fixation-related bacteria, symbiotic fungi, and saprotrophic fungi. Peruque was in favor of multiple fungi (*Russula* and *Inocybe*) to gain nutrition, while 87MX5-1.7 preferred a specific domain of fungi (*Tuber*) and nitrogen fixation-related bacteria (*Bradyrhizobia*) to form beneficial symbiosis. Moreover, the variation in pathogens indicated a potential risk of Fusarium patch and snow molds in pecan orchards with 87MX5-1.7 as a rootstock, while canker and black foot disease should be paid special attention in pecan orchards with Peruque as a rootstock. These findings underscore the importance of considering rootstock genotype selection in pecan orchard management, as it can directly impact soil microbial dynamics, nutrient cycling, and disease resistance. Furthermore, while the rhizosphere microbiomes were primarily influenced by rootstock genotype and geographical locations, certain components of the microbiomes form stable core microbiomes (including symbiotroph and pathogen) within the pecan rhizosphere. These core microbiomes should be recognized as key microbial constituents crucial for pecan growth and health. In further study, more rootstock genotypes should be investigated to expand the database of pecan rhizosphere microbes, which will provide a guideline for optimizing pecan production and promote soil health and sustainability in orchard ecosystems.

## Data Availability

The datasets presented in this study can be found in online repositories. The names of the repository/repositories and accession number(s) can be found in the article/Supplementary material.

## Footnotes

^1^https://mothur.org/wiki/miseq_sop/

## Supplementary material

Supplementary Figure S1 Relative abundance of fungal taxonomy at the class **(A)**, order **(B)**, and family **(C)** levels, and bacterial taxonomy at the class **(D)**, order **(E)**, and family **(F)** levels. The fungal class, order, and family with abundance below 5%, 3%, and 5% were defined as other separately. The bacterial class, order, and family with abundance below 3%, 5%, and 5% were defined as other separately.

Supplementary Figure S2 The fungal trophic mode **(A)** and ecological guilds **(B)** relative abundances. The individual trophic mode was then merged into three categories: pathotroph **(C)**, saprotroph **(D)**, and symbiotroph **(E)** in both pecan root and rhizosphere soil. The trophic mode, including PAT, will be calculated as pathotroph, with SAP and SYM being treated similarly. PAT=Pathotroph, SAP=Saprotroph, and SYM=symbiotroph. PAR = parasite. Different lowercase letters above the columns in each graph indicate a significant difference among the means by Duncan’s test (*p* < 0.05), and NS represents no significant difference among the means (*p* > 0.05).

Supplementary Figure S3 The composition of fungal groups overlapped in both root and rhizosphere soil of two rootstocks **(A)**, in root and rhizosphere soil of 87MX5-1.7 **(B)**, and in root and rhizosphere soil of Peruque **(C)**.

Supplementary Figure S4 The abundances of functional bacterial groups in pecan root and rhizosphere soil. The counts of functional bacterial groups were transformed using Log10(counts+1) in R and then normalized (divided by means) in origin to generate the heatmap.

Supplementary Table S1 The plant height and diameter of 12 rootstocks (before grafting) and scion ‘Pawnee’ (after 10 years grafting).

Supplementary Table S2 Rhizosphere soil characteristics of C. illinoinensis cv ‘Pawnee’ with different rootstocks.
