# Supplementary material for: The rootstock genotype shapes the diversity of pecan (Carya illinoinensis) rhizosphere microbial community
Source: Front Microbiol. 2024 Oct 3;15:1461685. doi: 10.3389/fmicb.2024.1461685 (PMC11484272; doi:10.3389/fmicb.2024.1461685)
Supplement: Supplementary file 1 [file Image_1.PDF]

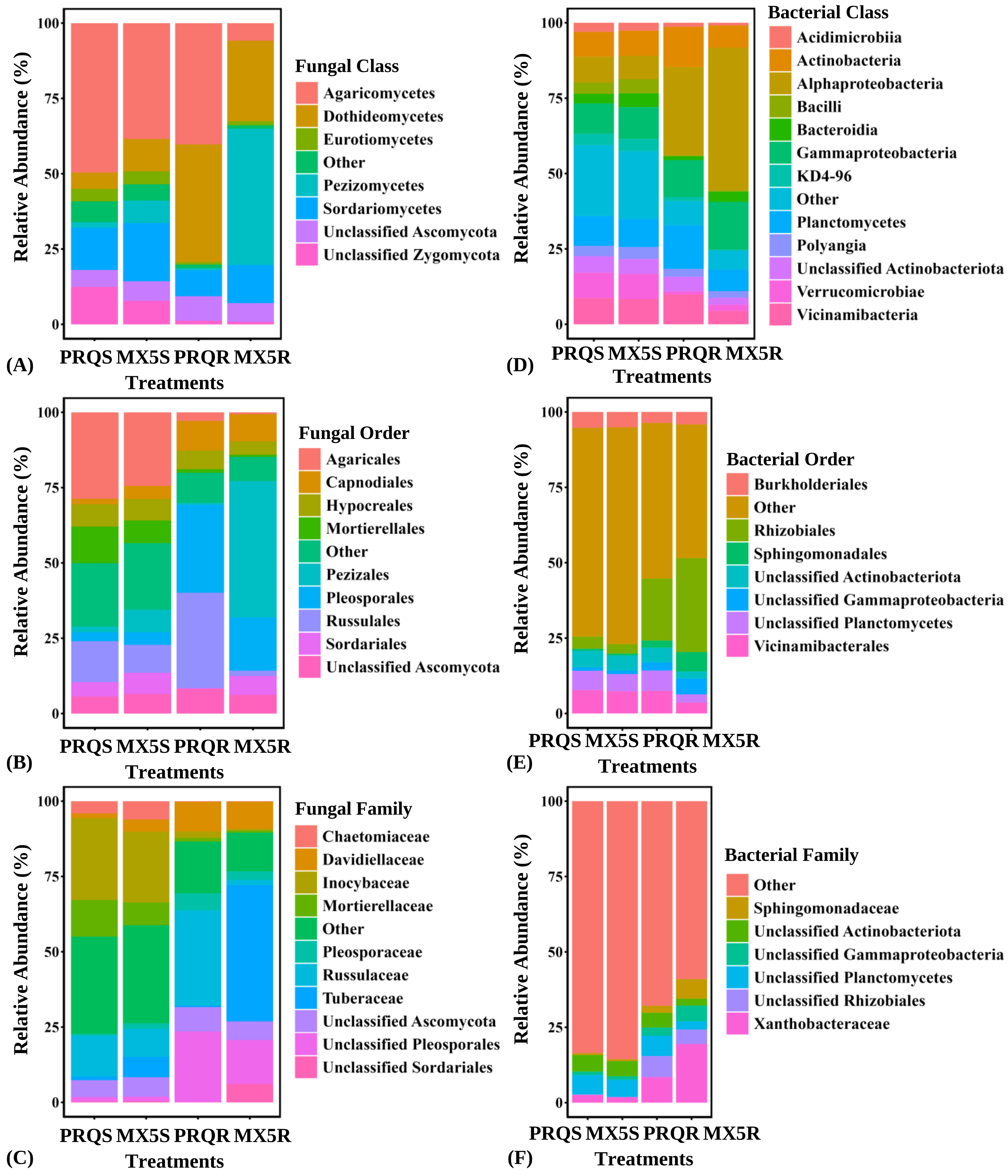

(A)
Relative Abundance (%)
0
25
50
75
100
PRQS MX5S PRQR MX5R
Treatments
Fungal Class
Agaricomycetes
Dothideomycetes
Eurotiomycetes
Other
Pezizomycetes
Sordariomycetes
Unclassified Ascomycota
Unclassified Zygomycota
(B)
Fungal Order
Agaricales
Capnodiales
Hypocreales
Mortierellales
Other
Pezizales
Pleosporales
Russulales
Sordariales
Unclassified Ascomycota
(C)
Fungal Family
Chaetomiaceae
Davidiellaceae
Inocybaceae
Mortierellaceae
Other
Pleosporaceae
Russulaceae
Tuberaceae
Unclassified Ascomycota
Unclassified Pleosporales
Unclassified Sordariales
(D)
Bacterial Class
Acidimicrobiia
Actinobacteria
Alphaproteobacteria
Bacilli
Bacteroidia
Gammaproteobacteria
KD4-96
Other
Planctomycetes
Polyangia
Unclassified Actinobacteriota
Verrucomicrobiae
Vicinamibacteria
(E)
Bacterial Order
Burkholderiales
Other
Rhizobiales
Sphingomonadales
Unclassified Actinobacteriota
Unclassified Gammaproteobacteria
Unclassified Planctomycetes
Vicinamibacterales
(F)
Bacterial Family
Other
Sphingomonadaceae
Unclassified Actinobacteriota
Unclassified Gammaproteobacteria
Unclassified Planctomycetes
Unclassified Rhizobiales
Xanthobacteraceae

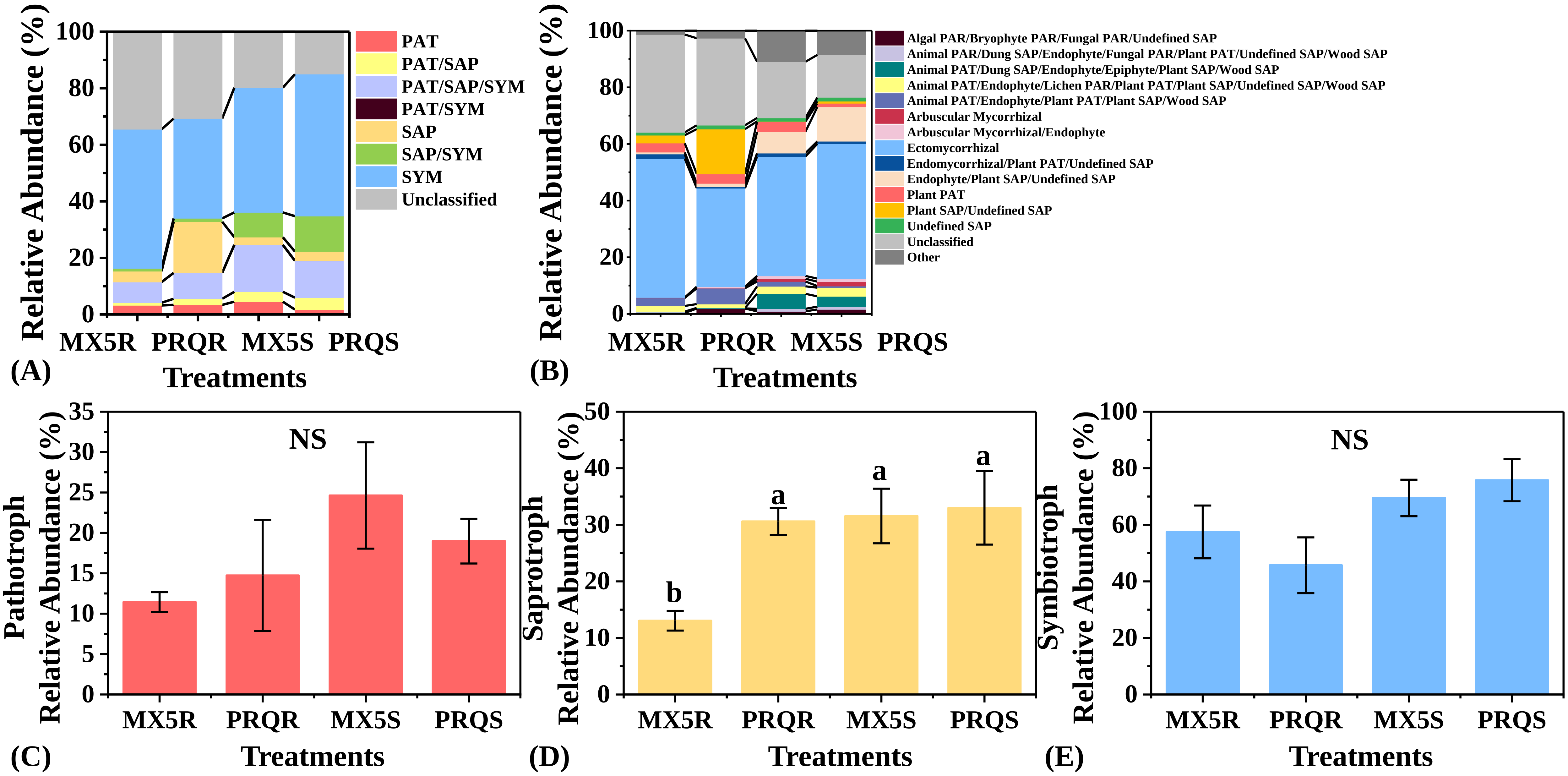

(A)
Relative Abundance (%)
Treatments
MX5R
PRQR
MX5S
PRQS
PAT
PAT/SAP
PAT/SAP/SYM
PAT/SYM
SAP
SAP/SYM
SYM
Unclassified
(B)
Relative Abundance (%)
Treatments
Algal PAR/Bryophyte PAR/Fungal PAR/Undefined SAP
Animal PAR/Dung SAP/Endophyte/Fungal PAR/Plant PAT/Undefined SAP/Wood SAP
Animal PAT/Dung SAP/Endophyte/Epiphyte/Plant SAP/Wood SAP
Animal PAT/Endophyte/Lichen PAR/Plant PAT/Plant SAP/Undefined SAP/Wood SAP
Animal PAT/Endophyte/Plant PAT/Plant SAP/Wood SAP
Arbuscular Mycorrhizal
Arbuscular Mycorrhizal/Endophyte
Ectomycorrhizal
Endomycorrhizal/Plant PAT/Undefined SAP
Endophyte/Plant SAP/Undefined SAP
Plant PAT
Plant SAP/Undefined SAP
Undefined SAP
Unclassified
Other
(C)
Pathotroph
Relative Abundance (%)
NS
Treatments
(D)
Saprotroph
Relative Abundance (%)
b
a
a
a
Treatments
(E)
Symbiotroph
Relative Abundance (%)
NS
Treatments

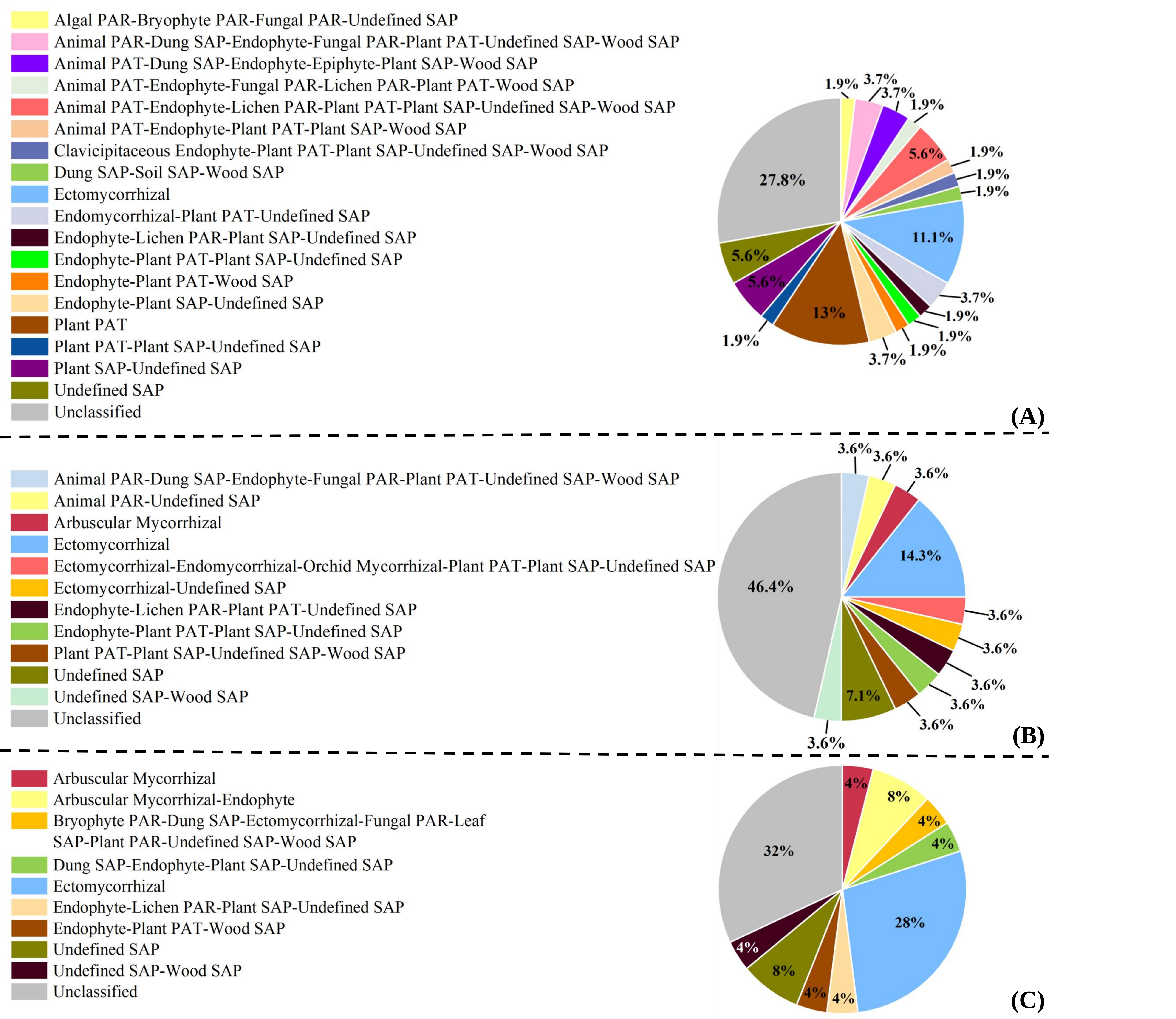
Algal PAR-Bryophyte PAR-Fungal PAR-Undefined SAP
Animal PAR-Dung SAP-Endophyte-Fungal PAR-Plant PAT-Undefined SAP-Wood SAP
Animal PAT-Dung SAP-Endophyte-Epiphyte-Plant SAP-Wood SAP
Animal PAT-Endophyte-Fungal PAR-Lichen PAR-Plant PAT-Wood SAP
Animal PAT-Endophyte-Lichen PAR-Plant PAT-Plant SAP-Undefined SAP-Wood SAP
Animal PAT-Endophyte-Plant PAT-Plant SAP-Wood SAP
Clavicipitaceous Endophyte-Plant PAT-Plant SAP-Undefined SAP-Wood SAP
Dung SAP-Soil SAP-Wood SAP
Ectomycorrhizal
Endomycorrhizal-Plant PAT-Undefined SAP
Endophyte-Lichen PAR-Plant SAP-Undefined SAP
Endophyte-Plant PAT-Plant SAP-Undefined SAP
Endophyte-Plant PAT-Wood SAP
Endophyte-Plant SAP-Undefined SAP
Plant PAT
Plant PAT-Plant SAP-Undefined SAP
Plant SAP-Undefined SAP
Undefined SAP
Unclassified
1.9%
3.7%
3.7%
1.9%
5.6%
1.9%
1.9%
1.9%
27.8%
11.1%
5.6%
5.6%
3.7%
1.9%
1.9%
1.9%
13%
1.9%
3.7%
(A)
Animal PAR-Dung SAP-Endophyte-Fungal PAR-Plant PAT-Undefined SAP-Wood SAP
Animal PAR-Undefined SAP
Arbuscular Mycorrhizal
Ectomycorrhizal
Ectomycorrhizal-Endomycorrhizal-Orchid Mycorrhizal-Plant PAT-Plant SAP-Undefined SAP
Ectomycorrhizal-Undefined SAP
Endophyte-Lichen PAR-Plant PAT-Undefined SAP
Endophyte-Plant PAT-Plant SAP-Undefined SAP
Plant PAT-Plant SAP-Undefined SAP-Wood SAP
Undefined SAP
Undefined SAP-Wood SAP
Unclassified
3.6%
3.6%
3.6%
14.3%
46.4%
3.6%
3.6%
3.6%
3.6%
7.1%
3.6%
3.6%
(B)
Arbuscular Mycorrhizal
Arbuscular Mycorrhizal-Endophyte
Bryophyte PAR-Dung SAP-Ectomycorrhizal-Fungal PAR-Leaf SAP-Plant PAR-Undefined SAP-Wood SAP
Dung SAP-Endophyte-Plant SAP-Undefined SAP
Ectomycorrhizal
Endophyte-Lichen PAR-Plant SAP-Undefined SAP
Endophyte-Plant PAT-Wood SAP
Undefined SAP
Undefined SAP-Wood SAP
Unclassified
4%
8%
4%
4%
32%
28%
4%
8%
4%
4%
(C)

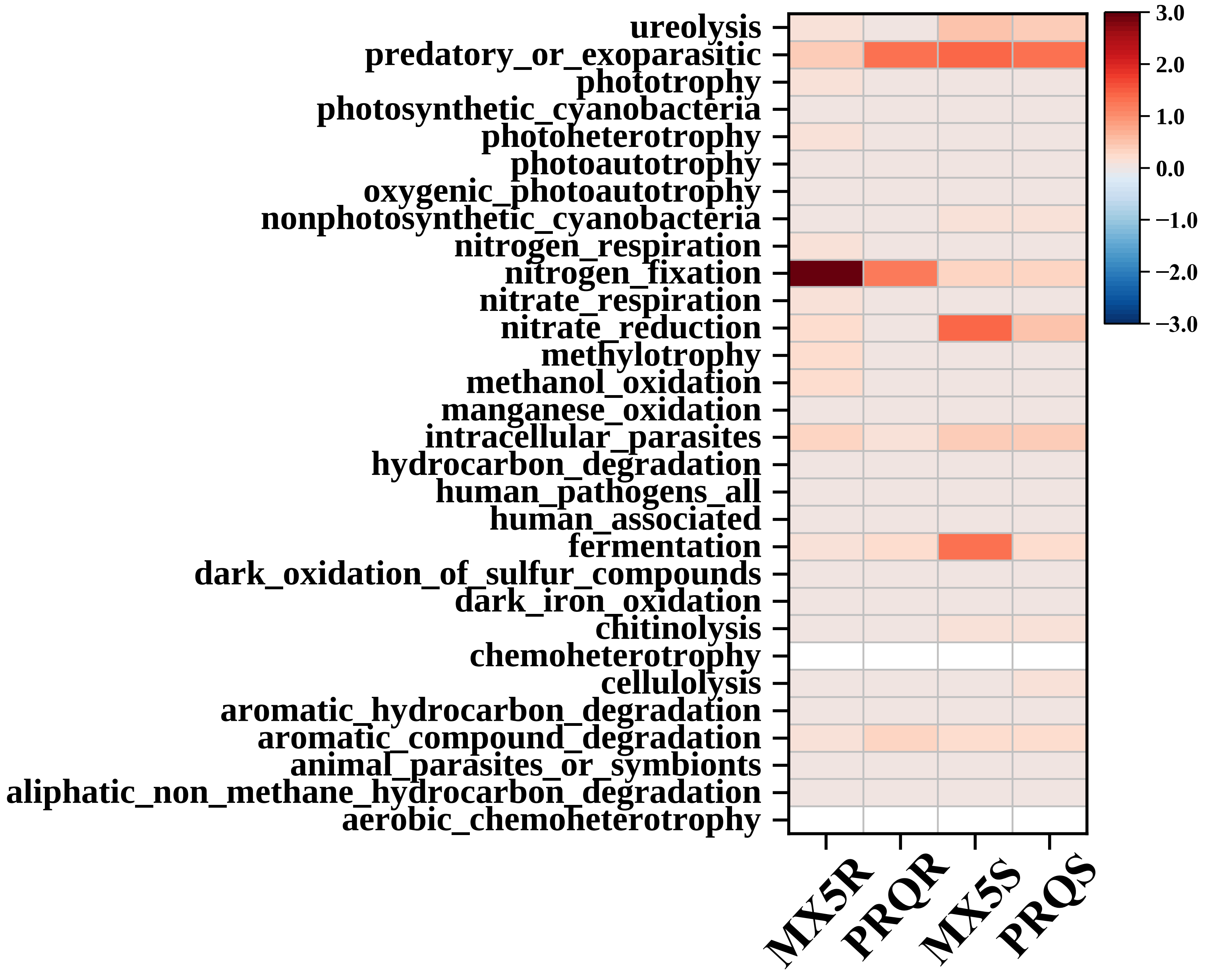
ureolysis
predatory_or_exoparasitic
phototrophy
photosynthetic_cyanobacteria
photoheterotrophy
photoautotrophy
oxygenic_photoautotrophy
nonphotosynthetic_cyanobacteria
nitrogen_respiration
nitrogen_fixation
nitrate_respiration
nitrate_reduction
methylotrophy
methanol_oxidation
manganese_oxidation
intracellular_parasites
hydrocarbon_degradation
human_pathogens_all
human_associated
fermentation
dark_oxidation_of_sulfur_compounds
dark_iron_oxidation
chitinolysis
chemoheterotrophy
cellulolysis
aromatic_hydrocarbon_degradation
aromatic_compound_degradation
animal_parasites_or_symbionts
aliphatic_non_methane_hydrocarbon_degradation
aerobic_chemoheterotrophy
MX5R
PRQR
MX5S
PRQS
3.0
2.0
1.0
0.0
−1.0
−2.0
−3.0
