# Supplementary material for: The rootstock genotype shapes the diversity of pecan (Carya illinoinensis) rhizosphere microbial community
Source: Front Microbiol. 2024 Oct 3;15:1461685. doi: 10.3389/fmicb.2024.1461685 (PMC11484272; doi:10.3389/fmicb.2024.1461685)
Supplement: Supplementary file 2 [file Table_1.docx]

**The rootstock genotype shapes the diversity of pecan (*Carya illinoinensis*) rhizosphere microbial community**

Supplementary Material

**Table S1** The plant height and diameter of 12 rootstocks (before grafting) and scion 'Pawnee' (after 10 years grafting)

**Table S2** Rhizosphere soil characteristics of *C. illinoinensis* cv 'Pawnee' with different rootstocks

**Figure S1** Relative abundance of fungal taxonomy at the class (A), order (B), and family (C) levels, and bacterial taxonomy at the class (D), order (E), and family (F) levels. The fungal class, order, and family with abundance below 5%, 3%, and 5% were defined as other separately. The bacterial class, order, and family with abundance below 3%, 5%, and 5% were defined as other separately.

**Figure S2** The fungal trophic mode (A) and ecological guilds (B) relative abundances. The individual trophic mode was then merged into three categories: pathotroph (C), saprotroph (D), and symbiotroph (E) in both pecan root and rhizosphere soil. The trophic mode, including PAT, will be calculated as pathotroph, with SAP and SYM being treated similarly. PAT=Pathotroph, SAP=Saprotroph, and SYM=symbiotroph. PAR = parasite. Different lowercase letters above the columns in each graph indicate a significant difference among the means by Duncan's test (*P* < 0.05), and NS represents no significant difference among the means (*P* > 0.05).

**Figure S3** The composition of fungal groups overlapped in both root and rhizosphere soil of two rootstocks (A), in root and rhizosphere soil of 87MX5-1.7 (B), and in root and rhizosphere soil of Peruque (C).

**Figure S4** The abundances of functional bacterial groups in pecan root and rhizosphere soil. The counts of functional bacterial groups were transformed using Log_10_(counts+1) in R and then normalized (divided by means) in origin to generate the heatmap.
